# Lyme Disease and Pregnancy

**DOI:** 10.1155/S1064744995000755

**Published:** 1995

**Authors:** James M. Alexander, Susan M. Cox

**Affiliations:** Department of Obstetrics and Gynecology University of Texas Southavestern Medical Center 5323 Harry Hines Boulevard Dallas TX 75235-9032 USA

## Abstract

Lyme disease is the most commonly transmitted vector-borne disease in the United States, with many regions of the country at risk. Like other spirochete-borne infections, Lyme disease progresses in stages, making diagnosis in the early stages of the illness and prompt treatment important for cure. An early diagnosis is made difficult by the less-than-ideal serologic tests and the varied clinical presentations of the disease. Although Lyme disease has been reported in pregnancy, the transmission rate to the fetus and potential harmful effects are largely unknown. This review discusses the diagnosis, clinical course, and treatment of Lyme disease with an emphasis on the pregnant patient.


Infectious Diseases in Obstetrics and Gynecology 3:256–261 (1995)
© 1996 Wiley-Liss, Inc.

### Lyme Disease and Pregnancy

**James M. Alexander and Susan M. Cox**
*Department of Obstetrics and Gynecology, University of Texas Southwestern Medical Center, Dallas, TX*

ABSTRACT

**Lyme disease is the most commonly transmitted vector-borne disease in the United States, with many regions of the country at risk. Like other spirochete-borne infections, Lyme disease progresses in stages, making diagnosis in the early stages of the illness and prompt treatment important for cure. An early diagnosis is made difficult by the less-than-ideal serologic tests and the varied clinical presentations of the disease. Although Lyme disease has been reported in pregnancy, the transmission rate to the fetus and potential harmful effects are largely unknown. This review discusses the diagnosis, clinical course, and treatment of Lyme disease with an emphasis on the pregnant patient.** © 1996 Wiley-Liss, Inc.

KEY WORDS
Erythema chronicum migrans, arthritis, ticks, neonatal infection, borrelial infection

Lyme disease, originally called Lyme arthritis, was first recognized in 1975 based on observations of geographic clustering of affected children in Lyme, CT.[1] These children were thought to have juvenile rheumatoid arthritis; however, an unusual skin lesion, erythema chronicum migrans (ECM), was often observed prior to the development of arthritis. The clustering of cases along with the heralding lesion suggested an infectious etiology of Lyme disease. Furthermore, the cases were more common in rural areas during the summer months, suggesting an arthropod vector. Epidemiologic studies of patients reporting ECM suggested the *Ixodes* tick, commonly known as the deer tick, as this vector. In 1982, Burgdorfer et al.[2] were able to isolate a previously unknown spirochete from the *I. dammini* tick. This spirochete is now known to be the etiologic agent that causes Lyme disease.

Clinically, Lyme disease is similar to other borrelial infections, most notably syphilis, in that it involves multiple organ systems and progresses in stages. The first report of the maternal-fetal transmission of Lyme disease in 1985 and subsequent case reports provide evidence that transplacental passage of the spirochete can result in fetal infection.[3–5] Some authors have suggested an increase in congenital malformations due to Lyme disease; however, this effect has not been proved conclusively to date. Because of the potential adverse fetal outcome and possible long-term maternal complications, it is important to understand the etiology, diagnosis, and treatment of Lyme disease. Furthermore, it appears that early treatment prevents most long-term complications in the mother and likely decreases adverse effects in the fetus.

### EPIDEMIOLOGY

The Centers for Disease Control (CDC) reports that, currently, Lyme disease is the most common vector-borne disease in the United States, with an estimated 1,500 cases annually. Although reported throughout the country, cases have been primarily clustered in three geographic areas: the Northeast (Massachusetts to Maryland), the Midwest (Wisconsin to Minnesota), and the West (Oregon to California).[6] Overseas, 1,000 new cases per year are re-

Address correspondence/reprint requests to Dr. James M. Alexander, Department of Obstetrics and Gynecology, University of Texas Southwestern Medical Center, 5323 Harry Hines Boulevard, Dallas, TX 75235-9032.

Received December 22, 1995
Accepted March 25, 1996
Review Article ported from Europe and additional cases are reported from the Soviet Union, China, Japan, and Australia.[7] The disease is usually transmitted during the warm months, primarily May and June. There has been a dramatic increase in the number of Lyme disease cases over the last decade for several reasons: an increase in the deer population, especially in the East; an influx of people into rural areas increasing exposure to disease-bearing ticks; and an increased recognition of Lyme disease by health care professionals.

### Vector

Lyme disease is caused by the spirochete *Borrelia burgdorferi* that is transmitted by the *Ixodes* tick (more commonly known as the deer tick). These vectors include *I. dammini* in the Northeast and Midwest, *I. pacificus* in the western states, *I. ricinus* in Europe, and *I. persulcatus* in Asia. In addition, *Amblyomma americanum* (known as the Lone Star tick) has been identified as a vector in Louisiana and Texas.[8] During its 2-year life cycle, the *Ixodes* tick undergoes 3 stages: larva, nymph, and adult. The adult female lays thousands of eggs during the spring which hatch during the late spring and early summer. The larva feed on the white-footed mouse, which is almost universally infected with *B. burgdorferi* in the summer months, resulting in transmission to the larval tick. The larva becomes dormant in the fall and winter, followed by molting in the spring which results in a nymph. The nymph then feeds on the deer mouse or other animals, including humans. If the nymph is infected with *B. burgdorferi*, vertical transmission (and human infection) can occur. In the fall, the nymph molts into the adult tick and attaches to a deer. Mating between adult ticks occurs, the female lays her eggs, and the cycle starts over.

### Clinical Manifestations

Like other syphilitic diseases, Lyme disease progresses in stages. Asbrink and Hovmark[9] described 3 states that correspond to the time course of disease progression. Stage I is identified by the characteristic skin lesion of Lyme disease, ECM, and nonspecific flu-like symptoms with regional lymphadenopathy. Weeks to months later, stage II develops with signs and symptoms of disseminated infection and multiorgan involvement such as cardiac or neurological abnormalities, musculoskeletal symptoms, or intermittent arthritis. Months to years later, stage III develops with chronic manifestations of disseminated disease (Table 1). Although this classification system is useful in describing the course of Lyme disease, there is much overlap in these stages, making a precise classification difficult.

ECM, the hallmark of Lyme disease, is seen in 60–80% of patients. Classically, the lesion starts as a small erythematous plaque or macule and then expands to a diameter of approximately 15 cm (range of 3–68 cm) with an area of centralized clearing.[10] The rash, typically seen on the thigh, groin, or axilla, may be associated with pruritis or a burning sensation. The rash lasts 3–4 weeks (range of 1 day to 14 months). In 50% of patients, multiple lesions develop. The primary lesion is usually associated with low-grade fever, flu-like symptoms (migratory arthralgias, myalgias, and headache), and regional lymphadenopathy.[11] Within days or weeks, the *B. burgdorferi* spirochete may spread to secondary sites through the patient's lymph and blood. During this disseminated (second) stage of Lyme disease, the neurologic, joint, and cardiovascular systems are primarily affected.

The most common neurologic complication of Lyme disease is headache, but more severe problems can occur. Within 4 weeks of exposure, 10–15% of patients exhibit a triad of neurologic symptoms: meningitis, cerebral-nerve palsy, and peripheral radiculopathies.[12] The meningitis is characterized by severe headaches with neck stiffness but without a Kernig's or Brudzinski's sign. A spinal tap typically reveals a lymphocytic pleocytosis (~100 WBC/mm$^3$) with minimally elevated protein and normal glucose.[12,13] One-half of the cases of meningitis are associated with a mild encephalitis with deficits in concentration, lethargy, and emotional lability. Cranial-nerve palsies, most commonly involving the facial nerve (Bell's palsy), may be seen concurrently with meningitis in as many as 15% of these patients.[13] Other nerve palsies involve the third and sixth cranial nerves. Also seen are peripheral and sensory radiculopathies which may include severe neuritic pain, dysthesias, focal weakness, and areflexia.[12,13]

Arthralgias and myalgias are commonly seen early in the disease. Approximately 60% of untreated patients experience brief attacks of asymmetric, oligoarticular arthritis. The arthritis usually affects the large joints, especially the knee. In ap-

TABLE 1. Common manifestations of Lyme disease by stage[a]

| System | Early infection: Localized (stage I) | Early infection: Disseminated (stage II) | Late infection: Persistent (stage III) |
|---|---|---|---|
| Skin | Erythema migrans | Secondary annular lesions, diffuse erythema | Acrodermatitis chronica atrophicans |
| Musculoskeletal | | Migratory joint pains, brief arthritis attacks | Prolonged arthritis attacks, chronic arthritis |
| Neurological | | Meningitis, Bell's palsy, motor or sensory radiculopathies | Chronic encephalomyelitis, spastic paraparesis, ataxic gait, subtle mental disorders |
| Lymphatic | Regional lymphadenopathy | Generalized lymphadenopathy, splenomegaly | |
| Heart | | Atrioventricular nodal block, myopericarditis, pancarditis | |
| Constitutional symptoms | Minor complaints | Severe malaise and fatigue | Chronic fatigue |

[a]The stages in this table provide a guideline for the timing of the manifestations of disease; however, the stages can be variable.

proximately 10% of these patients, chronic arthritis develops, resulting in erosion of the cartilage and bone and permanent joint disability. Interestingly, patients with HLA $DR_2$ and HLA $DR_4$ are more likely to be affected by arthritis, suggesting an altered immune response with autoimmune destruction triggered by the spirochete.[14] The synovial fluid reveals an elevated leukocyte count (range of 2,000–11,000) and a predominance of polymorphonuclear leukocytes. Immune complexes are also present, further suggesting an autoimmune-type phenomenon that results in joint destruction.[15]

In 4–10% of the patients with Lyme disease, cardiac complications develop 3–6 weeks after the onset of early symptoms.[16] The hallmark cardiac lesion is heart block which varies from first degree to complete. Usually, the conduction deficit is short lived (1–2 weeks), requiring only temporary pacing, if any. In a few patients, more diffuse cardiac involvement is seen, resulting in acute myocarditis, mild left ventricular dysfunction, and, rarely, cardiomegaly with fatal pancarditis.

The chronic manifestations of disseminated disease are well described. Chronic arthritis, which tends to affect one joint or a few large joints, especially the knee, can result in destruction of bone and cartilage and permanent joint disability. Syndromes of the central and peripheral nervous systems usually involve distal paresthesias or radiculopathy. In addition, subtle symptoms of central nervous system involvement can be seen with memory loss, somnolence, and behavioral changes. The most severe late nervous-system involvement is a progressive encephalomyelitis with spastic disparesis, bladder dysfunction, ataxia, cranial-nerve deficits, and dementia. A late skin manifestation of Lyme disease, acrodermatitis chronica atrophicans, which has also been well described, can lead to atrophy of the skin.

### PREGNANCY

Since Lyme disease was first described, there has been concern about the transmission of *B. burgdorferi* across the placenta with adverse fetal effects, as is seen with other spirochete diseases such as syphilis and relapsing fever. Isolated cases of the transplacental passage of *B. burgdorferi*, the first in 1985 by Schlesinger and colleagues,[17] have been documented. In that case, spirochetes were found in multiple organ systems in an infant who died shortly after birth from congenital heart disease. The mother, who had ECM early in pregnancy, did not receive antimicrobial therapy. Weber et al.[18] reported a case of Lyme disease in a woman treated with a subtherapeutic course of penicillin. The pregnancy progressed to term, but the infant died within 24 h of birth of respiratory failure, probably from antenatal brain damage. *B. burgdorferi* was isolated from the liver and brain of the infant. One other case of stillbirth in which the spirochete was isolated from multiple organs has been described.[19] These cases confirm that transplacental passage of the spirochete with fetal infection occurs; however, it is unclear if Lyme disease was the actual cause of fetal death.

A retrospective review of 19 cases of women diagnosed with Lyme disease in pregnancy revealed 5 infants with adverse fetal outcomes; however, none of the outcomes appeared related.[20] This review did not show a teratogenic effect of Lyme disease on the fetus. Another report of 17 women who had documented first-trimester infections showed 2 abnormal pregnancy outcomes: a spontaneous abortion and an infant with syndactyly. Neither case could be attributed to Lyme disease.[21] Three studies were carried out on cord-blood serology and the relationship of *B. burgdorferi*-specific antibody to the incidence of congenital malformations. Williams et al.[22] surveyed 463 infants, 282 from an endemic area and 181 from a nonendemic area, and found no association between the presence of antibody to *B. burgdorferi* and congenital malformations. Likewise, Nadal et al.[23] surveyed 1,416 mother/infant pairs and found a prevalence of positive serology of approximately 1%. Of these 12 infants, 1 born to a mother who had active Lyme disease during pregnancy had an isolated ventriculoseptal defect. In the 11 infants with elevated cord IgG titers, 6 had abnormal neonatal courses: 2 with hyperbilirubinemia, 1 with muscular hypotonia, 1 small-for-gestational age, 1 with microcephaly, and 1 with supraventricular tachycardia. All 6 were normal at 8 months of age. The authors concluded that there was no association of positive serology with adverse neonatal outcomes. Strobino et al.[24] prospectively studied just over 2,000 women in an endemic area and found positive serologies in 7.1%. As in the 2 previous studies, no correlation was seen between positive serology and congenital malformation, although the authors felt the results to be inconclusive because of the small number of cases. Furthermore, no increased risk of fetal death or low birth weight was seen despite the presence of positive serology.

In summary, while Lyme disease can be transmitted to the fetus, the incidence of transplacental passage is unknown. Several case reports have associated the *B. burgdorferi* organism with fetal infection, but this relationship is incompletely understood and fetal infection may be a preventable outcome with adequate treatment. Large surveys of women at risk in endemic areas have shown a low prevalence of seropositivity. In general, the pregnancy outcome in these women is similar to the general population. Because of the low incidence of Lyme disease, even in endemic areas, studies to date have not conclusively ruled out an adverse effect of Lyme disease on fetal outcome. For this reason, close observation of women at risk is recommended.

### DIAGNOSIS

The clinical diagnosis of Lyme disease is difficult because of the multiple manifestations of the disease and the infrequent recall of a tick bite. ECM, the hallmark lesion of Lyme disease, may not be present in 20–40% of patients. Although 3 stages of disease have been described, they are variable and overlap in the time of onset. Commercially available serologic tests can be helpful in establishing the diagnosis. The most commonly used and widely available tests are the immunofluorescent assay (IFA) and enzyme-linked immunosorbent assay (ELISA), with the more sensitive and specific Western blot used to confirm a positive result.[25–27] Paired serum samples can be obtained, with a rise in titer of the assay suggestive of disease, but monitoring the antibody titers is required over weeks to months. More helpful is the detection of IgM antibodies (usually within 2–4 weeks of exposure), followed by IgG antibodies (usually 6 weeks after exposure). The IgG antibody titer will typically rise higher than the IgM and persist for months or even years.[26]

One obvious limitation of serologic testing is the development of signs and symptoms of the disease prior to development of an antibody response, resulting in a false-negative result. In addition, if antibiotic treatment is initiated immediately, an antibody response may never develop, rendering serologic testing useless. Both IFA and ELISA methods are associated with false-positive results in the setting of syphilis, juvenile rheumatoid arthritis, systemic lupus erythematosus, and endocarditis.[25] The sensitivity and specificity of these tests, which vary widely from laboratory to laboratory, are dependent on when the test is obtained in relation to acquisition of the infection.[28] A further complication is the presence of the IgG antibody for months or years after an infection, making it difficult to determine if the current infection is primary or secondary. The Western blot assay, which is more sen-

TABLE 2. Treatment of Lyme disease during pregnancy[a]

| Disease manifestations | Regimen |
|---|---|
| ECM | Amoxicillin, 500 mg p.o. g.i.d. for 10 days (extended to 30 days with persistent symptoms) |
| | PCN allergy: erythromycin, 1 g orally/day in divided doses for 10–30 days |
| Carditis | Penicillin G, 200,000–300,000 units/kg/day (up to 20 million units/day) IV for 10 days *or* |
| | Ceftriaxone, 2 g/day for 10 days |
| | Prednisone, 1–2 mg/kg/day (if no response in first 24 h, temporary pacemaker for complete heart block) |
| Neurologic abnormalities | |
| Meningitis | Same as carditis |
| Isolated cranial-nerve palsy | Same as ECM |
| Motor deficits | Same as ECM (may require 7–8 weeks of therapy for persistent symptoms) |
| Arthritis | Amoxicillin and probenecid, 500 mg each orally g.i.d. for 30 days *or* |
| | Ceftriaxone, 2 g IV daily for 14 days *or* |
| | Penicillin G, 300,000 units q 4 h IV for 14 days |

[a]Adapted from Steere.[27]

sitive and specific than the IFA or ELISA, is currently used as a confirmatory test in many laboratories. As with the IFA and ELISA, the Western blot results vary widely between laboratories, with no standardization of results. The CDC is expected to issue a set of standardization guidelines soon. Because of the limitations of serologic testing, the current recommendation is to diagnose Lyme disease based on clinical and epidemiologic criteria and use serologic tests as adjuncts to the diagnosis.

### PREVENTION AND TREATMENT

It has been well documented that the early treatment of Lyme disease greatly reduces the later manifestations of disseminated infection.[29–32] The prevention of Lyme disease involves avoiding tick-infested areas, wearing appropriate clothing, and using proper insect repellent.[33] Examining the body for ticks and promptly removing them are important. Saving the tick may be useful for later identification. Future preventive measures may include a vaccine that is currently being studied.

Table 2 lists the treatment regimens for Lyme disease in pregnancy. Several caveats are important to consider in treating Lyme disease during pregnancy. While effective in treating Lyme disease and recommended as first-line treatment in non-pregnant patients, doxycycline is contraindicated in pregnancy and not included in Table 2. IV ceftriaxone, once daily, can be used as first-line therapy in a patient with neurologic symptoms or in a patient with difficulty in taking oral penicillin (which usually requires 6 divided doses/day). In a penicillin-allergic patient, chloramphenicol for 14 days is effective. The recommended duration of therapy may not be adequate for stage III joint or neurologic disease. A Jarisch-Herxheimer reaction, which may result in significant hypotension and fever, has been described.

### CONCLUSIONS

As the prevalence of Lyme disease has increased, the concern has grown about the effect of Lyme disease on the fetus of an infected mother. As discussed in this review, cases have been reported of transplacental passage of the spirochete, resulting in fetal infection and possibly death. However, the incidence of transplacental passage of Lyme disease and the actual risk of neonatal morbidity and mortality are currently unknown. Clearly, the early recognition and treatment of patients with Lyme disease decrease the risks of long-term complications, but the benefit to the fetus of early maternal treatment is unknown. Although serology is helpful after the first 3–4 weeks of infection, a clinical suspicion of disease and the recognition of signs and symptoms are the most important tools in establishing an early diagnosis. There is not enough information available to recommend specific fetal evaluation or intervention if the mother is infected with Lyme disease. The current recommendations emphasize close examination of the newborn for signs of intrapartum infection.

### REFERENCES

1. Steere AC, Malawista SE, Snydman DR, et al.: Lyme arthritis: An epidemic of oligoarticular arthritis in children and adults in three Connecticut communities. Arthritis Rheum 20:7–17, 1977.
2. Burgdorfer W, Barbour AG, Hayes SF, Benach JL, Grunwaldt E, David JP: Lyme disease—A tick-borne spirochetosis? Science 216:1317–1319, 1982.
3. Schlesinger PA, Duray PH, Burke BA, Steere AC, Stillman MT: Maternal-fetal transmission of the Lyme disease spirochete, *Borrelia burgdorferi.* Ann Intern Med 103:67–69, 1985.
4. Weber K, Bratzke HJ, Neubert U, Wilske B, Duray PH: *Borrelia burgdorferi* in a newborn despite oral penicillin for Lyme borreliosis during pregnancy. Pediatr Infect Dis 7:286–289, 1988.
5. Markowitz LE, Steere SC, Benach JL, Slade JD, Broome CV: Lyme disease during pregnancy. JAMA 255:3394–3396, 1986.
6. Tsai TS, Bailey RE, Moore PS: National surveillance of Lyme disease, 1987–1988. Conn Med 53:324–326, 1989.
7. Stanek G, Pletschette M, Flamm H, et al.: European Lyme borreliosis. Ann NY Acad Sci 539:274–282, 1988.
8. Steere AC, Malawista SE: Cases of Lyme disease in the United States: Locations correlated with distribution of *Ixodes dammini.* Ann Intern Med 91:730–733, 1979.
9. Asbrink E, Hovmark A: Early and late cutaneous manifestations of *Ixodes*-borne borreliosis (erythema migrans borreliosis, Lyme borreliosis). Ann NY Acad Sci 539:4–15, 1988.
10. Steere AC, Bartenhagen NH, Craft JE, et al.: The early clinical manifestations of Lyme disease. Ann Intern Med 99:76–82, 1983.
11. Steere SC, Taylor E, Wilson ML, Levine JF, Spielman A: Longitudinal assessment of the clinical and epidemiological features of Lyme disease in a defined population. J Infect Dis 154:295–300, 1986.
12. Pachner AR, Steere AC: The triad of neurologic manifestations of Lyme disease: Meningitis, cranial neuritis, and radiculoneuritis. Neurology 3:47–53, 1985.
13. Reik L, Steere AC, Bartenhagen NH, Shope RE, Malawista SE: Neurologic abnormalities of Lyme disease. Medicine (Baltimore) 58:281–294, 1979.
14. Steere AC, Feld J, Winchester R: Association of chronic Lyme arthritis with increased frequencies of DR4 and 3 (abstract). Arthritis Rheum 31:S98, 1988.
15. Hardin JA, Steere AC, Malawista SE: Immune complexes and the evolution of Lyme arthritis: Dissemination and localization of abnormal Clq binding activity. N Engl J Med 301:1358–1563, 1979.
16. Steere AC, Batsford WP, Weinberg M, et al.: Lyme carditis: Cardiac abnormalities of Lyme disease. Ann Intern Med 93:8–16, 1980.
17. Schlesinger PA, Duray PH, Burker BA, Steere AC, Stillman MT: Maternal-fetal transmission of the Lyme disease spirochete, *Borrelia burgdorferi.* Ann Internal Med 102:67–69, 1985.
18. Weber K, Bratzke H-J, Neubert U, Wilske B, Duray PH: *Borrelia burgdorferi* in a newborn despite oral penicillin for Lyme borreliosis during pregnancy. Pediatr Infect Dis 7:286–289, 1988.
19. MacDonald AB, Benach JL, Burgdorfer W: Stillbirth following maternal Lyme disease. NY State J Med Nov:615–616, 1987.
20. Markowitz LE, Steere AC, Benach JL, Slade JD, Broome CV: Lyme disease during pregnancy. JAMA 255:3394–3396, 1986.
21. Ciesielski CA, Russell H, Johnson S, et al.: Prospective study of pregnancy outcome in women with Lyme disease (abstract). 27th ICAAC, 1987.
22. Williams CL, Benach JL, Curran AS, Spierling P, Medici F: Lyme disease during pregnancy: A cord blood serosurvey. Ann NY Acad Sci 539:504, 1988.
23. Nadal D, Hunziker UA, Bucher HU, Hitzig WH, Duc G: Infants born to mothers with antibodies against *Borrelia burgdorferi* at delivery. Eur J Pediatr 148:426–427, 1989.
24. Strobino BA, Williams CL, Abid S, Chalson R, Spierling P: Lyme disease and pregnancy outcome: A prospective study of two thousand prenatal patients. Am J Obstet Gynecol 169:367–374, 1993.
25. Mangarelli LA, Meegan JM, Anderson JF, Chappell WA: Comparison of an indirect fluorescent-antibody test with an enzyme-linked immunosorbent assay for serological studies of Lyme disease. J Clin Microbiol 20:181–184, 1984.
26. Craft JE, Grodzicki RL, Steere AC: Antibody response in Lyme disease: Evaluation of diagnostic tests. J Infect Dis 149:789–795, 1984.
27. Steere AC: Lyme disease. N Engl J Med 321:586–596, 1989.
28. Hedberg CW, Osterholm MT, MacDonald KL, White KE: An interlaboratory study of antibody to *Borrelia burgdorferi.* J Infect Dis 155:1325–1327, 1987.
29. Steere AC, Malawista SE, Newman JH, Spieler PN, Bartenhagen NH: Antibiotic therapy in Lyme disease. Ann Intern Med 93:1–8, 1980.
30. Steere AC, Hutchinson GJ, Rahn DW, et al.: Treatment of the early manifestations of Lyme disease. Ann Intern Med 99:22–26, 1983.
31. Steere AC, Pachner AR, Malawista SE: Neurologic abnormalities of Lyme disease: Successful treatment with high-dose intravenous penicillin. Ann Intern Med 99: 767–772, 1983.
32. Steere AC, Greene J, Schoen RT, et al.: Successful parenteral penicillin therapy of established Lyme arthritis. N Engl J Med 312:869–874, 1985.
33. Stafford KC: Lyme disease prevention: Personal protection and prospects for tick control. Conn Med 53:347–351, 1988.
